# Why are healthcare professionals leaving NHS roles? A secondary analysis of routinely collected data

**DOI:** 10.1186/s12960-024-00951-8

**Published:** 2024-09-20

**Authors:** Alison Leary, Elaine Maxwell, Rebecca Myers, Geoffrey Punshon

**Affiliations:** 1 London South Bank University, London, UK; 2 The Queen’s Nursing Institute, London, UK

## Abstract

**Background:**

Much policy attention focuses on increasing the supply of workers in the English NHS but there has been less attention paid to the rise in leavers. This paper seeks to explore how existing data sets can illuminate the decision-making of leavers and inform actions that could mitigate this.

**Method:**

Secondary analysis of routinely collected data from 79 workforce projects in the UK (*n* = 46 339 participants) over a 4-year (2019–2023) period was undertaken. Free text data we extracted and analysed using content analysis, sentiment analysis and text mining. Inclusion criteria were those who stated they had resigned, had confirmed retirement date, and had secured employment elsewhere either within or without the sector but had not yet resigned. Exclusion criteria were those who had not indicated they were leaving or indicated intention to leave. These findings were then compared with themes from Herzberg’s work hygiene theory and Hoffat and Woods’s professional practice environment theory.

**Results:**

Multiple reasons were given for leaving. Findings were congruent with Herzberg’s two factor work hygiene theory and demonstrate that leavers are driven by the inability to meet their intrinsic motivation to practice according to their professional standards as much as by terms and conditions. Leavers describe suboptimal professional practice environments which produce obstacles to achieving their work objectives and leaving their intrinsic motivation frustrated.

**Conclusion:**

Whilst reasons for leaving differ between people, there is a relationship between intrinsic motivation (why they want to do the job) and the conditions in which they try to do the job. This study suggests that looking beyond the primary reason for leaving given in the national dataset could identify how the practice environment influences the decision.

## What is already known on this topic

There are currently retention issues within the National Health Service, with a high rate of leavers. Routinely collected NHS data on reasons for leaving NHS roles are broad (such as retirement), singular and often missing.

## What this study adds

This study provides insight and an increased understanding of the complexity/multiplicity of reasons for leaving NHS roles. It demonstrates the importance of the practice environment on the decision to leave as well as factors like pay and conditions.

## How this study might affect research, practice or policy

This study can be used to improve data quality around why people leave NHS jobs. It adds understanding of the multiplicity of reasons people leave which can be addressed by employers and policy makers. It identified that there are extrinsic and intrinsic factors that need to be addressed to retain staff. The findings demonstrate leavers becoming frustrated by threats to their intrinsic motivation. Whilst pay is a very real impediment for some, terms and conditions are often confirmation that the organisation does not recognise the value of the work the person is motivated to do. This is also demonstrated by the way work is organised, the way performance is evaluated and the relationships between staff groups. However, the mechanisms that influence staff decision-making on leaving are under studied and largely focus on the individual member of staff rather than the context in which they work.

## Strengths and limitations of the study

This study gives insight into the multiplicity of reasons for leaving and how addressing the practice environment might help retention.

This is a non-stratified, secondary analysis of a convenience sample, it does give some insight into the multiplicity of reasons employees give for leaving NHS and a research study designed to explore these issues would offer more insight and generalisability.

## Introduction

There is a growing demand for healthcare, in England alone with almost 7.7 million people waiting for treatment or elective surgery in December 2023 and there is also a growing need for care in the community [1]. In the National Health Service (NHS) in England data on leavers are collected by employers and collated centrally. Leavers data are categorised with 15 different categories such as retirement, resignation, dismissal, redundancy and death. There are sub categories for example, resignation has sub categories such as promotion, work life balance but the largest group in this and other sub categories in “unknown” [2].

There is much policy attention to increasing the supply of workers in the English NHS [3] but there has been less attention paid to the significant rise in leavers, in part because of the lack of detailed understanding about why staff leave. The UK’s NHS has a wealth of data from leavers that is under analysed. This paper seeks to explore how existing data sets, collated as part of 79 different commissioned workforce projects, can illuminate the decision-making of leavers and inform actions that could mitigate this.

Against the backdrop of growing demand for healthcare [4, 5] there is an unmet staffing demand in the UK NHS. Prior to the COVID-19 pandemic, there was a modest growth of staff in the NHS in England (18 587 more than the previous year) but this was set against a reported 100 000 vacancies. The growth was patchy across professions, with 3% growth in ambulance staff, scientific staff, and medical staff (although this varied across specialities) but only a 1% increase in nurses [6]. This modest growth may be in part due to a lack of supply as a result of poor workforce forecasting^.^ In addition to a low growth rate, the average intention to leave in the studies conducted by the authors has been rising. In May 2021 it rose sharply from an average of 19% (consistent since 2005) to 60% and has remained at this level in 2023. There has been a rise in those leaving their profession before retirement age [7]. This is thought to cause collateral damage as the workforce policy focuses on the supply of less experienced and qualified workers [8], which in turn increases greater job demand [9, 10] on remaining staff, burnout, and further leavers, creating a vicious circle. The Covid-19 pandemic further accelerated this [11].

In the nationally collected leavers data [2] only one reason is allocated to each leaver. As well as NHS employer data, several other bodies commission pulse and longer structured surveys into NHS workers across the UK, either to model the workforce or understand specific issues. Although the surveys vary in design, all surveys ask respondents’ intention and action regarding their workplace as closed questions but with the opportunity to enter free text data. These qualitative data are rarely examined collectively, and the aim of the study was to seek common patterns or themes in this data.

## Methods

Secondary analysis of routinely collected data from 79 workforce projects (*n* = 46 339 participants *n* = range 310–3090) over a 4-year (2019–2023) period was performed.

The workforce projects included ranged from local pulse surveys for NHS Trusts (23), through to large workforce modelling projects for specialisms that were UK wide (7). There were also UK large cross-sectional surveys of groups such as District Nurses (28) commissioned by charities such as the Queens Nursing Institute and national workforce/service evaluations for specialisms such as diagnostics or specific treatment areas such as cancer (21). All data were collected via different types of survey with free text options. The majority (56) was from national (UK wide) projects. All of these projects were commissioned but delivered and analysed independently. Inclusion criteria were those who stated they had resigned, had confirmed retirement plan/retirement date, or had secured employment elsewhere either within or without the sector but had not yet resigned. Exclusion criteria were those who had not indicated they were leaving or only indicated intention to leave.

The free text was mined using RapidMiner for key phrases (for example ‘I have resigned’). Responses were then analysed by two researchers to establish concordance using Cohen’s Kappa for interrater reliability. An inductive approach was used to develop a codebook. Responses were initially read by one researcher to determine themes and then re-read to identify subthemes. The emergent codebook had 12 over-arching themes which reached at least 50 occurrences. In addition, an “other” category that had less than 50 responses, including emigration, retirement, specific personal and potentially identifiable circumstances and choosing not to renew contracts. Reliability was high between the two manual reviewers (Ƙ 0.89 unweighted).

Sentiment analysis was then applied to text and was found to be negative overall. These findings were then qualitatively compared with themes from Herzberg’s work hygiene theory [12] and Hoffart and Woods’s professional practice environment theory [13].

### Patient and public involvement

Patients and the public were not involved in any way in this study.

## Results

In total 1910 respondents from the datasets met the criteria for inclusion, of whom 1886 respondents completed the free text box with reasons for leaving (see Table 1). 24 respondents did not give any reason. 4579 reasons for leaving were analysed. Respondents could give more than one reason for leaving.

**Table 1:** Breakdown of respondents by profession and place of work

	Community	Hospital	Primary care	Other
Registered Nurse	460	1172	62	14
Support Workers and Nursing Associates	2	6	6	0
Physician	0	76	26	3
Allied Health Professional (AHP)	19	47	1	1
Pharmacists	8	2	1	0
Scientists and physiologists	0	4	0	0
Total	489	1307	96	18

**Fig. 1:**
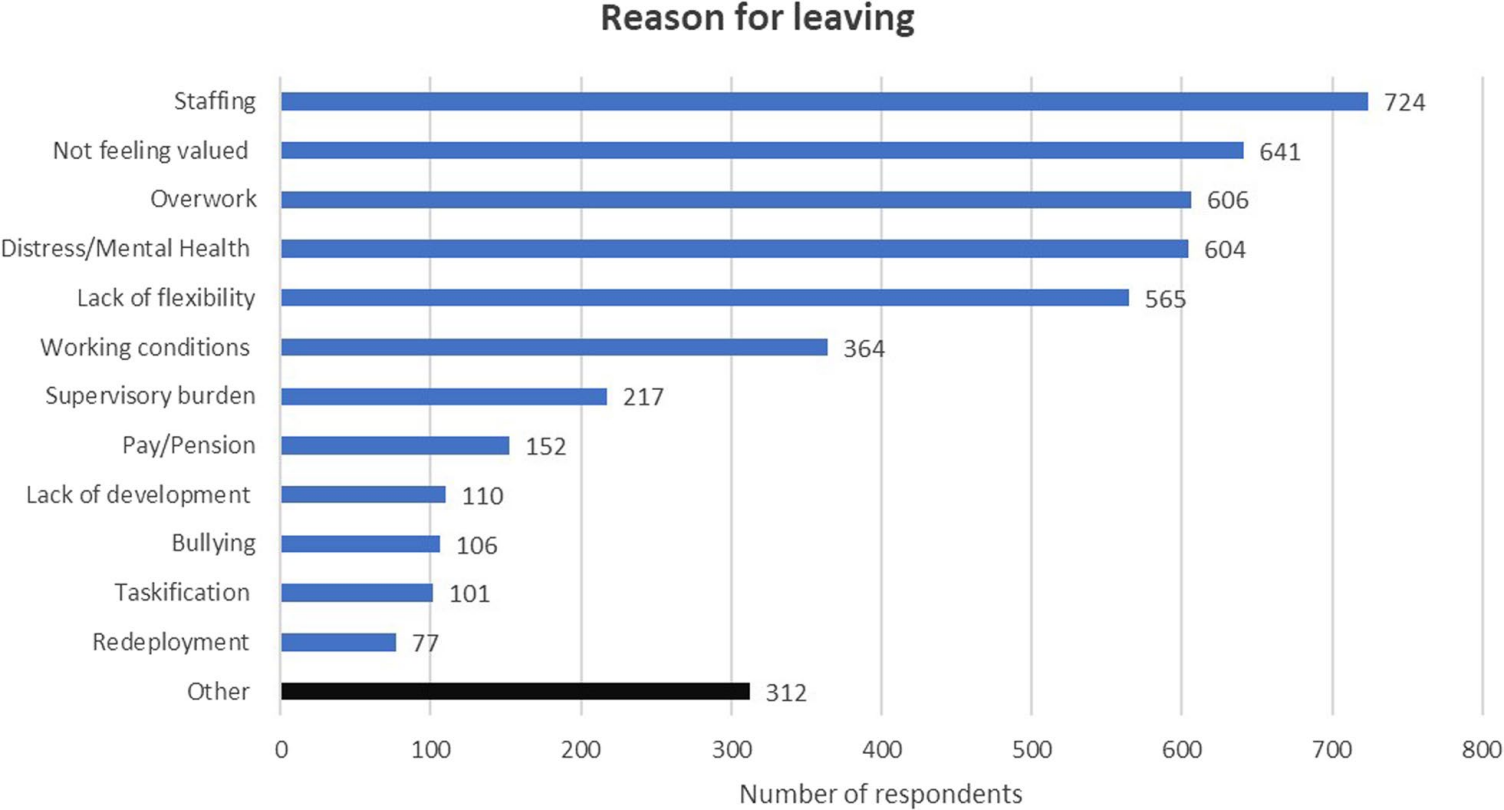
The frequency of response categories

Free text responses were assigned into 13 categories (see Fig. 1). Respondents often gave a primary reason for leaving “I’m retiring but…” and then gave additional reasons to qualify the decision with a mean of 2.2 reasons for leaving per person (range 1–6). At first, the relationships between each of the sets of reasons are not clear. Following a critical realist ontology [14], the authors sought to explore the underlying relationships that are not explicitly visible from the responses by mapping them onto two models of work satisfaction that have been used widely in healthcare [15, 16]. Herzberg et al. two factor model asserts the satisfaction is determined by two separate sets of conditions: intrinsic motivation and extrinsic work hygiene conditions [12]. Intrinsic satisfaction is enhanced by feeling valued and a feeling of achievement and productivity. Extrinsic satisfaction is exacerbated by working conditions, pay, job security, organisational policies, and status. Neither intrinsic nor extrinsic factors have primacy, and both contribute to what Hoffart and Woods [13] call the professional practice environment, which they describe as having five key components: professional values, professional relationships, professional patient care delivery system, management approach and compensation and rewards structure.

The nature of the professional practice environments in healthcare services has been shown to be associated with better retention of staff [17]. Karaferis et al. found a correlation between recognition of healthcare workers in Greece and work motivation [18]. Similarly, Wilson (2015) surveyed allied healthcare professionals (AHP) in Australia and found a significant correlation between feeling competent to do the job, recognition for doing the job, advancement opportunities, autonomy, feelings of worthwhile accomplishment, communication and support from the manager and intention to leave [19]. Veld and Van de Voorde found that positive work environments enhanced commitment as well as retention. In particular they found that those who felt a ‘social’ relationship with their workplace were more committed than those who perceived it as an economic relationship [20].

### Intrinsic motivation

The data is this study shows that threats to intrinsic motivation is the driving force in decisions to leave. Intrinsic motivation is linked to sense of self actualisation [21], or a feeling of having done a good job. Most of the leavers expressed reasons that reflect a working environment that challenged their sense of self actualisation.

“I go home knowing I have done a bad job as we don’t have enough staff” (Band 6 RN)

In some cases, the cognitive dissonance of providing care that resulted in the opposite of their intrinsic motivations made staff feel ill:

“The ward is really unsafe, and I feel sick at the thought of coming to work” (Band 4 Support Worker)

This experience can lead to moral distress [22–24]. In an attempt to resolve this, staff work harder and longer, compromising their work life balance and this eventually results in burnout.

“Just really, really tired and have decided to retire. I won’t be coming back” (Physician, Primary Care)

“Every Sunday afternoon I spend catching up on admin and emails when I’d rather spend it with my grandchildren, so I am retiring” (Band 7 AHP)

“We are just given more to do, the better you are, the more you are given to do and managers don’t care how tired we are” (Band 7 RN)

The subcategories of reasons for leaving identified in the data suggest that these threats come both from the organisational values and from the working conditions. The relationship between the two can be complex and we suggest that in the context of poor work hygiene conditions, it is the mechanism of the professional practice environment that influences the outcome, reflecting Rafferty et al. finding that the way work is organised is as important as the number of staff or educational level of nurses on both patient outcomes and staff satisfaction [25]. Thus, the professional practice environment is critical to maintaining intrinsic motivation and thereby job satisfaction.

### Elements of professional practice environment

#### Professional values

Professions establish their work boundaries based on values that are reflected in their professional jurisdiction [26]. These recognise the theoretical basis for their work and thus their skill and contribution. Intrinsic motivation is challenged in practice environment where these values are disregarded. The data in this study showed that there were multiple ways in which staff experience this.

Staff reported that organisations do not recognise the unique contribution of different occupational groups:

“We have to fill in for everyone, ward clerk is off, nurse will do it, housekeeper off, nurse will do it, we are not valued” (Band 5 RN)

This reflected the taskification of care, where care is divided into a series of tasks rather than a holistic approach. Managerial focus is on the completion of tasks (regardless of who completed them) and value that distinct groups of staff bring to the overall outcome was disregarded.

“Technology has made the role as DN [District Nurse] a more task orientated role rather than a holistic autonomous role it used to be, tick boxes are now the driving force for quality outcomes, rather than patient focused, individualised care planning.” (Band 7 RN)

“I’m no longer giving good care, it’s just about targets, that’s all managers care about” (Band 6 RN)

“I don’t deal with people anymore just tasks the joy has gone out of my work” (Physician Primary Care)

Failure to recognise the ‘gestalt’ of professional practice, and the resulting division of work into discrete tasks means that important but invisible professional work is not recognised. As it is not recognised, the educational or developmental work to support and develop professional practice is replaced by specific task-based education:

“Have been a CNS for ten years but there is no funding to develop any further or undertake my masters, being left behind” (Band 6 RN)

“There is nowhere to go, no development opportunities” (Band 5 RN)

“No career prospects here, keep being turned down for courses” (Band 6 AHP)

Failure to recognise professional values is also demonstrated by the way in which staff are deployed. Mandated redeployment during the COVID-19 pandemic where staff numbers were measured but there was a lack of appreciation of the complexity and risk and its impact on staff. This was cited as a reason for leaving by registered nurses:

“The pandemic was horrendous I was redeployed but also still had my own caseload” (Band 7 RN)

However, redeployment of staff (particularly nurses) has been and remains a constant feature, independent of pandemics:

“I often have to move wards and it causes me to be anxious” (Band 5 RN)

“We often have to fill in on the wards, once I was put in charge and I have not worked on a ward for ten years, its disrespectful to us and our patients” (Band 6 RN)

### Professional relationships

Many studies have shown that social support helps employees to effectively mitigate workplace stress [27] and is associated with higher job satisfaction and reduced turnover intentions [28]. Laschinger et al. (2014) proposed that this is particularly so when there is a collective (as opposed to individual) perception that the work environment has good inter professional relationships [29]. Perceptions of poor relationships and poor leadership have been linked with intention to leave in UK [30], Canada [31, 32] and other European countries [33].

Poor relationships, described as bullying, were explicitly given as reasons for leaving in the data. Consideration of the subcategories showed this included poor group dynamics that were not managed:

“Cliques at work are really nasty, I don’t like it here” (Band 5 AHP)

Sometimes managers were said to be complicit:

“Bullies are friends with managers, no point reporting it” (Band 5 RN)

“I work with bullies, they bully the staff and the patients, no one cares” (Band 3 Support Worker)

Bullying also related to the way in which managers handled performance management:

“Everything I do is wrong according to my manager, but they don’t help me to learn” (Band 3 Support Worker)

### Professional patient care delivery system

Professional practice environments have (intentionally or unintentionally designed) care delivery systems that can enhance or impede professional values. In the data (lack of) staffing was by far the most common reason given for leaving. This led staff to become anxious about the safety of their practice:

“I was the only nurse on for sixty patients on two wards overnight, the staffing is unsafe, I have resigned” (Band 5 RN)

“The workload is too much, I have too many patients, I don’t feel safe at work” (Primary Care Physician)

“Can’t recruit staff they may have to close service when I have gone” (Community Pharmacist)

Where staff had additional duties, such as the supervision of unregistered staff, students, and new second-level roles, they had less time to practice directly with patients and this challenged their motivation. When compounded with a shortage of peers, staff felt that they were not doing any of the work to a satisfactory standard:

“Most shifts I have to support and supervise support workers, students, international colleagues waiting for PIN and sometimes volunteers. I don’t have time to supervise or teach” (Band 5 RN)

“Our support workers are fantastic, but I can’t supervise them all and it makes me anxious. I know we are missing things, so I have decided to leave” (Band 6 RN)

Technology is often promoted as a tool to improve practice environments. However poor implementation, or systems that do not pay attention to professional values can make things worse not better:

“Due to [scheduling platform] often poor function, things are being missed/duplicated leading to time wasting and mistakes. One of the reasons I am retiring now.” (Band 6 RN)

“The work seems pointless; the IT never works” (Band 7 clinical scientist)

Leadership is a key determinant of a practice environment, and local managers are critical in how staff perceive the organisation’s values, regardless of corporate mission statements. Individual teams may differ dependent on their line managers:

“Managers don’t value what we do for patients.” (Band 6 AHP)

“My manager doesn’t understand our work at all, they are not a nurse, they don’t understand nursing” (Band 6 RN)

### Compensation and rewards structure

Whilst Herzberg et al. propose that job satisfaction is determined by intrinsic motivation, and professional practice environments may enhance or impede this, a distinct concept of dissatisfaction with terms and conditions is often given as a reason for leaving [12]. Pay and pension ranked 9th of the 13 categories in terms of frequency and its subcategories of pay and pension relate to the lack of affordability of continuing to do the job:

“I love what I do, I just can’t afford to do it anymore” (Band 6 RN)

“I have two kids and third on the way, I can’t afford to do this job now” (Band 4 Support Worker)

“I am retiring. The pension issues make it impossible for me to stay” (Consultant Physician)

Ironically, promotion (which might be expected to be perceived as a recognition of value and confirm intrinsic motivation) often comes with a reduction in take home pay, sending mixed messages on value:

*“I was promoted but lost unsocial hours for more responsibility, more work for less money doesn’t make me feel valued”* (Band 7 RN).

This is also apparent in employers' responses to requests for flexible working, where the member of staff would be doing the same work, with the same skill mix but was told this would be on a lower pay grade:

*“Asked to work part time but told policy is no part time for band 7. Offered a band 5 job/secondment so I have got a job at [charity] for band 7 pay”.* (Band 7 RN).

There was no regard given to the role of retaining experienced staff in supporting the team:

“I’m a carer for my parents and my manager refused flexible working as it would have a negative effect on the team” (Band 3 Support Worker)

## Discussion

Raw data about who leaves their jobs are abundant in healthcare services but translating these data into useful information requires careful analysis. Both high and low rates of turnover can be problematic for patient safety, indicating either widespread staff dissatisfaction or conversely elevated levels of complacency and failure to reflect and challenge.

The data in this study support a model of leavers becoming frustrated by threats to their intrinsic motivation. Whilst pay is a very real impediment for some, terms and conditions are often confirmation that the organisation does not recognise the value of the work the person is motivated to do. This is also demonstrated by the way work is organised, the way performance is evaluated and the relationships between staff groups. However, the mechanisms that influence staff decision-making on leaving are under studied and largely focus on the individual member of staff rather than the context in which they work.

Many of the current surveys and exit interview tools used to gain insight into why people leave only consider extrinsic factors. Whilst ensuring these are good is important, they are not sufficient on their own and missing data on intrinsic factors may lead to simplistic or spurious conclusions When there is a mismatch in the rating of extrinsic and intrinsic factors, the consequences may be counter intuitive. Indeed, many staff work in sub optimal conditions but can feel high job satisfaction if their intrinsic job needs are met. Conversely, if intrinsic motivation is not met staff may leave to seek more motivating work, whilst others may remain but lack motivation despite good pay and conditions. Staff motivation to either remain in their post or to leave is therefore complex and is influenced by multiple and potentially conflicting factors.

Operational failures were associated with low patient satisfaction scores, poor quality and safety outcomes, and poor nurse job outcomes, and those associations were partly accounted for by clinical work environments.

The relationship between the number of registered healthcare professionals and both patient outcomes and organisational outcomes has been well established [34–40]. However, there is an increasing gap between the optimal number of staff and the actual numbers [41]. Attempts to fill the workforce gap in high-income countries have included increased numbers of unregistered staff and international recruitment of registered nurses (despite the global shortfall of registered nurses being most acute in low and middle-income countries) [42]. Other solutions include the creation of new posts with lower levels of educational preparation (second-level roles such as Nursing Associates, Physician and Anaesthetic Associates). These new roles have mixed evidence. Twigg et al. found that adding a second-level nurse in Australia increased patient harms [43]. On the other hand, Drennan et al. found that Physician Associates can provide a flexible clinical practitioner in secondary care without drawing from existing professions such as nurses [44]. Failure to manage both roles well in the UK has led concerns that new roles encroach on the existing role jurisdictions [45], increasing the dissatisfaction of existing staff. It also appears that expansion of the workforce in associate and supportive roles is adding to supervisory burden, first described by Menzies Lyth in the 1960s [46]. The increased use of volunteers in roles previously undertaken by clinical staff [47] and technology to replace the need for human input are, as yet, untested [48]. Attrition in the healthcare and social care workforce has become a major concern in the UK. Although spending and activity are increasing, service provision is hampered by significant retention issues [49] understanding the multiplicity of reasons for leaving could significantly improve retention if addressed. Therefore, employers must pay attention to both extrinsic and intrinsic factors and should measure intrinsic satisfaction through the professional practice environment. Regular measurements or analysis of existing measures could identify trends that could be addressed before staff make the decision to leave.

It is possible to measure intrinsic factors through free text/semi-structured questions in instruments such as pulse surveys and that employers could use existing tools to monitor the professional practice environment on a regular basis to identify trends and also to address particular problems (largely a sense of feeling valued). An American study of 37 685 respondents surveyed at the start of the COVID pandemic found only 45% felt valued; Healthcare workers who felt highly valued had 8.3 times lower odds of burnout and 10.2 lower odds of intent to leave than those who did not feel valued at all [50]. This analysis shows that these data do exist, but employers need to utilise it in retaining staff.

## Data Availability

The datasets used and/or analysed during the current study are available from the corresponding author on reasonable request.
